# Supplementary figures and images for: Fatty acid profiles of highly migratory resources from the Southeastern Pacific Ocean, Chile: a potential tool for biochemical and nutritional traceability
Source: PeerJ. 2025 Mar 20;13:e19101. doi: 10.7717/peerj.19101 (PMC11930215; doi:10.7717/peerj.19101)

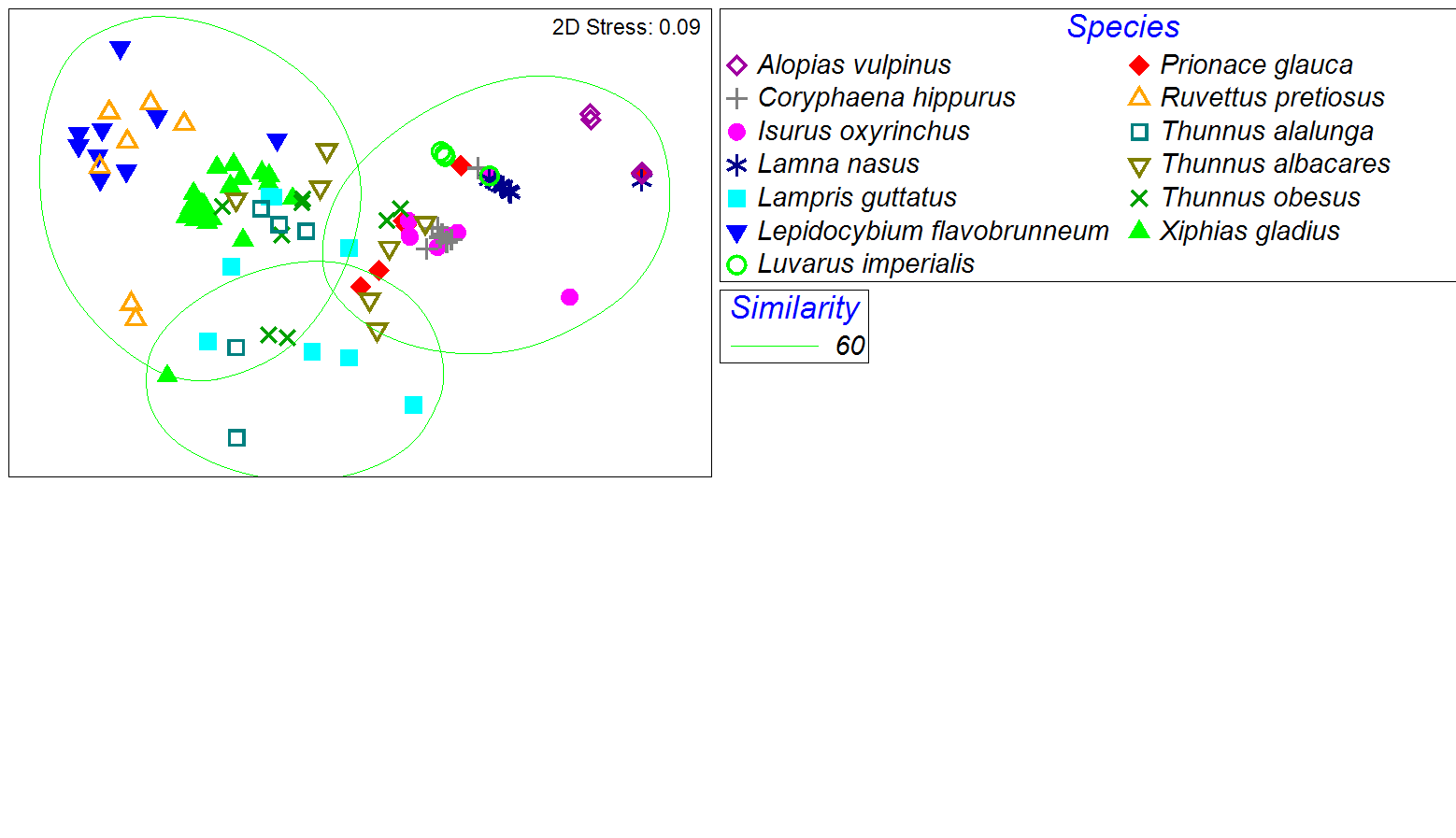

Supplement: Supplemental Information 1 — Different colors and forms represent the different species. [file peerj-13-19101-s001.png]
