# Supplementary material for: Fatty acid profiles of highly migratory resources from the Southeastern Pacific Ocean, Chile: a potential tool for biochemical and nutritional traceability
Source: PeerJ. 2025 Mar 20;13:e19101. doi: 10.7717/peerj.19101 (PMC11930215; doi:10.7717/peerj.19101)
Supplement: Supplemental Information 3 — *Comparisons: the following species were not included in the analysis, Makaira indica, Tetrapturus audax, Makaira indica, Sphyrna zygaena due to a lack of replication (only one specimen was analyzed). [file peerj-13-19101-s003.docx]

**Table S1.** Analysis of similarities (ANOSIM) of the muscle tissue of N=18 highly migratory species off the coast of Chile. Global statistical test (R= 0.761).

| **Pairwise Tests Groups** | **R Statistic** | **Significance**  **Level %** | | **Possible Perm.** | **Actual**  **Perm.** | |  |  |
| --- | --- | --- | --- | --- | --- | --- | --- | --- |
| *Xiphias gladius, Lepidocybium flavobrunneum* | 0.841 | 0.1 | | 94143280 | 999 | |  |  |
| *Xiphias gladius, Lampris guttatus* | 0.754 | 0.1 | | 23535820 | 999 | |  |  |
| *Xiphias gladius, Prionace glauca* | 0.951 | 0.1 | | 23535820 | 999 | |  |  |
| *Xiphias gladius, Isurus oxyrinchus* | 0.992 | 0.1 | | 23535820 | 999 | |  |  |
| *Xiphias gladius, Coryphaena hippurus* | 0.994 | 0.1 | | 23535820 | 999 | |  |  |
| *Xiphias gladius, Thunnus obesus* | 0.639 | 0.1 | | 23535820 | 999 | |  |  |
| *Xiphias gladius, Lamna nasus* | 1 | 0.1 | | 23535820 | 999 | |  |  |
| *Xiphias gladius, Ruvettus pretiosus* | 0.869 | 0.1 | | 5379616 | 999 | |  |  |
| *Xiphias gladius, Thunnus albacares* | 0.755 | 0.1 | | 5379616 | 999 | |  |  |
| *Xiphias gladius, Thunnus alalunga* | 0.661 | 0.1 | | 201376 | 999 | |  |  |
| *Xiphias gladius, Alopias vulpinus* | 1 | 0.1 | | 31465 | 999 | |  |  |
| *Xiphias gladius, Luvarus imperialis* | 0.994 | 0.1 | | 31465 | 999 | |  |  |
| *Xiphias gladius, Katsuwonus pelamis* | 0.999 | 0.2 | | 406 | 406 | |  |  |
| *Xiphias gladius, Gasterochisma melampus* | 0.908 | 0.5 | | 406 | 406 | |  |  |
| *Xiphias gladius, Makaira indica* | 0.585 | 7.1 | | 28 | 28 | |  |  |
| *Xiphias gladius, Tetrapturus audax* | 0.412 | 17.9 | | 28 | 28 | |  |  |
| *Xiphias gladius, Sphyrna zygaena* | 0.997 | 3.6 | | 28 | 28 | |  |  |
| *Lepidocybium flavobrunneum, Lampris guttatus* | 0.78 | 0.1 | | 24310 | 999 | |  |  |
| *Lepidocybium flavobrunneum, Prionace glauca* | 0.857 | 0.1 | | 24310 | 999 | |  |  |
| *Lepidocybium flavobrunneum, Isurus oxyrinchus* | 0.989 | 0.1 | | 24310 | 999 | |  |  |
| *Lepidocybium flavobrunneum, Coryphaena hippurus* | 0.993 | 0.1 | | 24310 | 999 | |  |  |
| *Lepidocybium flavobrunneum, Thunnus obesus* | 0.859 | 0.1 | | 24310 | 999 | |  |  |
| *Lepidocybium flavobrunneum, Lamna nasus* | 0.999 | 0.1 | | 24310 | 999 | |  |  |
| *Lepidocybium flavobrunneum, Ruvettus pretiosus* | 0.214 | 1.2 | | 11440 | 999 | |  |  |
| *Lepidocybium flavobrunneum, Thunnus albacares* | 0.894 | 0.1 | | 11440 | 999 | |  |  |
| *Lepidocybium flavobrunneum, Thunnus alalunga* | 0.807 | 0.2 | | 2002 | 999 | |  |  |
| *Lepidocybium flavobrunneum, Alopias vulpinus* | 1 | 0.1 | | 715 | 715 | |  |  |
| *Lepidocybium flavobrunneum, Luvarus imperialis* | 0.966 | 0.1 | | 715 | 715 | |  |  |
| *Lepidocybium flavobrunneum, Katsuwonus pelamis* | 0.994 | 1.8 | | 55 | 55 | |  |  |
| *Lepidocybium flavobrunneum, Gasterochisma melampus* | 0.204 | 27.3 | | 55 | 55 | |  |  |
| *Lepidocybium flavobrunneum, Makaira indica* | 0.802 | 10 | | 10 | 10 | |  |  |
| *Lepidocybium flavobrunneum, Tetrapturus audax* | 0.735 | 20 | | 10 | 10 | |  |  |
| *Lepidocybium flavobrunneum, Sphyrna zygaena* | 0.981 | 10 | | 10 | 10 | |  |  |
| *Lampris guttatus, Prionace glauca* | 0.472 | 0.1 | | 6435 | 999 | |  |  |
| *Lampris guttatus, Isurus oxyrinchus* | 0.777 | 0.1 | | 6435 | 999 | |  |  |
| *Lampris guttatus, Coryphaena hippurus* | 0.835 | 0.1 | | 6435 | 999 | |  |  |
| *Lampris guttatus, Thunnus obesus* | 0.299 | 1.5 | | 6435 | 999 | |  |  |
| *Lampris guttatus, Lamna nasus* | 0.932 | 0.1 | | 6435 | 999 | |  |  |
| *Lampris guttatus, Ruvettus pretiosus* | 0.752 | 0.1 | | 6435 | 999 | |  |  |
| *Lampris guttatus, Thunnus albacares* | 0.321 | 1.5 | | 6435 | 999 | |  |  |
| *Lampris guttatus, Thunnus alalunga* | 0.196 | 8.4 | | 1287 | 999 | |  |  |
| *Lampris guttatus, Alopias vulpinus* | 0.996 | 0.2 | | 495 | 495 | |  |  |
| *Lampris guttatus, Luvarus imperialis* | 0.746 | 0.2 | | 495 | 495 | |  |  |
| *Lampris guttatus, Katsuwonus pelamis* | 0.901 | 2.2 | | 45 | 45 | |  |  |
| *Lampris guttatus, Gasterochisma melampus* | 0.716 | 2.2 | | 45 | 45 | |  |  |
| *Lampris guttatus, Makaira indica* | -0.054 | 77.8 | | 9 | 9 | |  |  |
| *Lampris guttatus, Tetrapturus audax* | 0.089 | 44.4 | | 9 | 9 | |  |  |
| *Lampris guttatus, Sphyrna zygaena* | 0.804 | 11.1 | | 9 | 9 | |  |  |
| *Prionace glauca, Isurus oxyrinchus* | 0.138 | 8.6 | | 6435 | 999 | |  |  |
| *Prionace glauca, Coryphaena hippurus* | 0.365 | 0.1 | | 6435 | 999 | |  |  |
| *Prionace glauca, Thunnus obesus* | 0.45 | 0.1 | | 6435 | 999 | |  |  |
| *Prionace glauca, Lamna nasus* | 0.283 | 3 | | 6435 | 999 | |  |  |
| *Prionace glauca, Ruvettus pretiosus* | 0.819 | 0.1 | | 6435 | 999 | |  |  |
| *Prionace glauca, Thunnus albacares* | 0.288 | 1.1 | | 6435 | 999 | |  |  |
| *Prionace glauca, Thunnus alalunga* | 0.489 | 0.6 | | 1287 | 999 | |  |  |
| *Prionace glauca, Alopias vulpinus* | 0.068 | 21.8 | | 495 | 495 | |  |  |
| *Prionace glauca, Luvarus imperialis* | 0.037 | 33.7 | | 495 | 495 | |  |  |
| *Prionace glauca, Katsuwonus pelamis* | -0.056 | 57.8 | | 45 | 45 | |  |  |
| *Prionace glauca, Gasterochisma melampus* | 0.78 | 2.2 | | 45 | 45 | |  |  |
| *Prionace glauca, Makaira indica* | 0.33 | 11.1 | | 9 | 9 | |  |  |
| *Prionace glauca, Tetrapturus audax* | 0.384 | 11.1 | | 9 | 9 | |  |  |
| *Prionace glauca, Sphyrna zygaena* | -0.089 | 66.7 | | 9 | 9 | |  |  |
| *Isurus oxyrinchus, Coryphaena hippurus* | 0.11 | 4.7 | | 6435 | 999 | |  |  |
| *Isurus oxyrinchus, Thunnus obesus* | 0.621 | 0.1 | | 6435 | 999 | |  |  |
| *Isurus oxyrinchus, Lamna nasus* | 0.541 | 0.1 | | 6435 | 999 | |  |  |
| *Isurus oxyrinchus, Ruvettus pretiosus* | 1 | 0.1 | | 6435 | 999 | |  |  |
| *Isurus oxyrinchus, Thunnus albacares* | 0.379 | 0.1 | | 6435 | 999 | |  |  |
| *Isurus oxyrinchus, Thunnus alalunga* | 0.859 | 0.2 | | 1287 | 999 | |  |  |
| *Isurus oxyrinchus, Alopias vulpinus* | 0.967 | 0.2 | | 495 | 495 | |  |  |
| *Isurus oxyrinchus, Luvarus imperialis* | 0.474 | 0.2 | | 495 | 495 | |  |  |
| *Isurus oxyrinchus, Katsuwonus pelamis* | 0.591 | 6.7 | | 45 | 45 | |  |  |
| *Isurus oxyrinchus, Gasterochisma melampus* | 1 | 2.2 | | 45 | 45 | |  |  |
| *Isurus oxyrinchus, Makaira indica* | 0.902 | 11.1 | | 9 | 9 | |  |  |
| *Isurus oxyrinchus, Tetrapturus audax* | 0.964 | 11.1 | | 9 | 9 | |  |  |
| *Isurus oxyrinchus, Sphyrna zygaena* | 0.384 | 22.2 | | 9 | 9 | |  |  |
| *Coryphaena hippurus, Thunnus obesus* | 0.728 | 0.1 | | 6435 | 999 | |  |  |
| *Coryphaena hippurus, Lamna nasus* | 0.613 | 0.1 | | 6435 | 999 | |  |  |
| *Coryphaena hippurus, Ruvettus pretiosus* | 1 | 0.2 | | 6435 | 999 | |  |  |
| *Coryphaena hippurus, Thunnus albacares* | 0.496 | 0.1 | | 6435 | 999 | |  |  |
| *Coryphaena hippurus, Thunnus alalunga* | 0.897 | 0.1 | | 1287 | 999 | |  |  |
| *Coryphaena hippurus, Alopias vulpinus* | 1 | 0.2 | | 495 | 495 | |  |  |
| *Coryphaena hippurus, Luvarus imperialis* | 0.881 | 0.2 | | 495 | 495 | |  |  |
| *Coryphaena hippurus, Katsuwonus pelamis* | 0.772 | 4.4 | | 45 | 45 | |  |  |
| *Coryphaena hippurus, Gasterochisma melampus* | 1 | 2.2 | | 45 | 45 | |  |  |
| *Coryphaena hippurus, Makaira indica* | 1 | 11.1 | | 9 | 9 | |  |  |
| *Coryphaena hippurus, Tetrapturus audax* | 1 | 11.1 | | 9 | 9 | |  |  |
| *Coryphaena hippurus, Sphyrna zygaena* | 0.58 | 11.1 | | 9 | 9 | |  |  |
| *Thunnus obesus, Lamna nasus* | 0.874 | 0.1 | | 6435 | 999 | |  |  |
| *Thunnus obesus, Ruvettus pretiosus* | 0.94 | 0.1 | | 6435 | 999 | |  |  |
| *Thunnus obesus, Thunnus albacares* | 0.025 | 30.7 | | 6435 | 999 | |  |  |
| *Thunnus obesus, Thunnus alalunga* | 0.133 | 14.2 | | 1287 | 999 | |  |  |
| *Thunnus obesus, Alopias vulpinus* | 0.993 | 0.2 | | 495 | 495 | |  |  |
| *Thunnus obesus, Luvarus imperialis* | 0.623 | 0.2 | | 495 | 495 | |  |  |
| *Thunnus obesus, Katsuwonus pelamis* | 0.884 | 2.2 | | 45 | 45 | |  |  |
| *Thunnus obesus, Gasterochisma melampus* | 0.944 | 2.2 | | 45 | 45 | |  |  |
| *Thunnus obesus, Makaira indica* | -0.17 | 66.7 | | 9 | 9 | |  |  |
| *Thunnus obesus, Tetrapturus audax* | 0.036 | 66.7 | | 9 | 9 | |  |  |
| *Thunnus obesus, Sphyrna zygaena* | 0.768 | 11.1 | | 9 | 9 | |  |  |
| *Lamna nasus, Ruvettus pretiosus* | 1 | 0.1 | | 6435 | 999 | |  |  |
| *Lamna nasus, Thunnus albacares* | 0.717 | 0.1 | | 6435 | 999 | |  |  |
| *Lamna nasus, Thunnus alalunga* | 0.951 | 0.1 | | 1287 | 999 | |  |  |
| *Lamna nasus, Alopias vulpinus* | 0.658 | 0.2 | | 495 | 495 | |  |  |
| *Lamna nasus, Luvarus imperialis* | 0.412 | 3.6 | | 495 | 495 | |  |  |
| *Lamna nasus, Katsuwonus pelamis* | 0.181 | 11.1 | | 45 | 45 | |  |  |
| *Lamna nasus, Gasterochisma melampus* | 1 | 2.2 | | 45 | 45 | |  |  |
| *Lamna nasus, Makaira indica* | 1 | 11.1 | | 9 | 9 | |  |  |
| *Lamna nasus, Tetrapturus audax* | 1 | 11.1 | | 9 | 9 | |  |  |
| *Lamna nasus, Sphyrna zygaena* | 0.348 | 33.3 | | 9 | 9 | |  |  |
| *Ruvettus pretiosus, Thunnus albacares* | 0.91 | 0.2 | | 1716 | 999 | |  |  |
| *Ruvettus pretiosus, Thunnus alalunga* | 0.803 | 0.1 | | 792 | 792 | |  |  |
| *Ruvettus pretiosus, Alopias vulpinus* | 1 | 0.3 | | 330 | 330 | |  |  |
| *Ruvettus pretiosus, Luvarus imperialis* | 1 | 0.3 | | 330 | 330 | |  |  |
| *Ruvettus pretiosus, Katsuwonus pelamis* | 1 | 2.8 | | 36 | 36 | |  |  |
| *Ruvettus pretiosus, Gasterochisma melampus* | 0.156 | 19.4 | | 36 | 36 | |  |  |
| *Ruvettus pretiosus, Makaira indica* | 0.905 | 12.5 | | 8 | 8 | |  |  |
| *Ruvettus pretiosus, Tetrapturus audax* | 0.864 | 12.5 | | 8 | 8 | |  |  |
| *Ruvettus pretiosus, Sphyrna zygaena* | 1 | 12.5 | | 8 | 8 | |  |  |
| *Thunnus albacares, Thunnus alalunga* | 0.165 | 10.6 | | 792 | 792 | |  |  |
| *Thunnus albacares, Alopias vulpinus* | 0.974 | 0.3 | | 330 | 330 | |  |  |
| *Thunnus albacares, Luvarus imperialis* | 0.376 | 3.3 | | 330 | 330 | |  |  |
| *Thunnus albacares, Katsuwonus pelamis* | 0.604 | 2.8 | | 36 | 36 | |  |  |
| *Thunnus albacares, Gasterochisma melampus* | 0.916 | 2.8 | | 36 | 36 | |  |  |
| *Thunnus albacares, Makaira indica* | -0.252 | 87.5 | | 8 | 8 | |  |  |
| *Thunnus albacares, Tetrapturus audax* | -0.02 | 62.5 | | 8 | 8 | |  |  |
| *Thunnus albacares, Sphyrna zygaena* | 0.224 | 37.5 | | 8 | 8 | |  |  |
| *Thunnus alalunga, Alopias vulpinus* | 1 | 0.8 | | 126 | 126 | |  |  |
| *Thunnus alalunga, Luvarus imperialis* | 0.781 | 0.8 | | 126 | 126 | |  |  |
| *Thunnus alalunga, Katsuwonus pelamis* | 0.873 | 4.8 | | 21 | 21 | |  |  |
| *Thunnus alalunga, Gasterochisma melampus* | 0.673 | 4.8 | | 21 | 21 | |  |  |
| *Thunnus alalunga, Makaira indica* | -0.32 | 83.3 | | 6 | 6 | |  |  |
| *Thunnus alalunga, Tetrapturus audax* | -0.12 | 50 | | 6 | 6 | |  |  |
| *Thunnus alalunga, Sphyrna zygaena* | 0.72 | 16.7 | | 6 | 6 | |  |  |
| *Alopias vulpinus, Luvarus imperialis* | 1 | 2.9 | | 35 | 35 | |  |  |
| *Alopias vulpinus, Katsuwonus pelamis* | 0.357 | 26.7 | | 15 | 15 | |  |  |
| *Alopias vulpinus, Gasterochisma melampus* | 1 | 6.7 | | 15 | 15 | |  |  |
| *Alopias vulpinus, Makaira indica* | 1 | 20 | | 5 | 5 | |  |  |
| *Alopias vulpinus, Tetrapturus audax* | 1 | 20 | | 5 | 5 | |  |  |
| *Alopias vulpinus, Sphyrna zygaena* | 1 | 20 | | 5 | 5 | |  |  |
| *Luvarus imperialis, Katsuwonus pelamis* | 0.571 | 13.3 | | 15 | 15 | |  |  |
| *Luvarus imperialis, Gasterochisma melampus* | 1 | 6.7 | | 15 | 15 | |  |  |
| *Luvarus imperialis, Makaira indica* | 1 | 20 | | 5 | 5 | |  |  |
| *Luvarus imperialis, Tetrapturus audax* | 1 | 20 | | 5 | 5 | |  |  |
| *Luvarus imperialis, Sphyrna zygaena* | 0.25 | 40 | | 5 | 5 | |  |  |
| *Katsuwonus pelamis, Gasterochisma melampus* | 1 | 33.3 | | 3 | 3 | |  |  |
| *Katsuwonus pelamis, Makaira indica* | 1 | 33.3 | | 3 | 3 | |  |  |
| *Katsuwonus pelamis, Tetrapturus audax* | 1 | 33.3 | | 3 | 3 | |  |  |
| *Katsuwonus pelamis, Sphyrna zygaena* | 0 | 66.7 | | 3 | 3 | |  |  |
| *Gasterochisma melampus, Makaira indica* | 1 | 33.3 | | 3 | 3 | |  |  |
| *Gasterochisma melampus, Tetrapturus audax* | 1 | 33.3 | | 3 | 3 | |  |  |
| *Gasterochisma melampus, Sphyrna zygaena* | 1 | 33.3 | | 3 | 3 | |  |  |
|  |  | |  | | |  | |  |

*The species *Makaira indica*, *Tetrapturus audax* and *Sphyrna zygaena* could not be included in the analysis due to a lack of replication (only one specimen was analyzed).
